# Minimally Symptomatic Infection in an Ebola ‘Hotspot’: A Cross-Sectional Serosurvey

**DOI:** 10.1371/journal.pntd.0005087

**Published:** 2016-11-15

**Authors:** Eugene T. Richardson, J. Daniel Kelly, Mohamed Bailor Barrie, Annelies W. Mesman, Sahr Karku, Komba Quiwa, Regan H. Marsh, Songor Koedoyoma, Fodei Daboh, Kathryn P. Barron, Michael Grady, Elizabeth Tucker, Kerry L. Dierberg, George W. Rutherford, Michele Barry, James Holland Jones, Megan B. Murray, Paul E. Farmer

**Affiliations:** 1 Department of Anthropology, Stanford University, Stanford, United States of America; 2 Division of Global Health Equity, Brigham and Women’s Hospital, Boston, United States of America; 3 Partners In Health, Sierra Leone; 4 UCSF School of Medicine, San Francisco, United States of America; 5 Department of Global Health and Social Medicine, Harvard Medical School, Boston, United States of America; 6 Kono District Ebola Response Centre (DERC), Koidu, Sierra Leone; 7 Kono District Health Management Team (DHMT), Koidu, Sierra Leone; 8 UCSF Global Health Sciences, San Francisco, United States of America; 9 Stanford University School of Medicine, Stanford, United States of America; Tulane School of Public Health and Tropical Medicine, UNITED STATES

## Abstract

**Introduction:**

Evidence for minimally symptomatic Ebola virus (EBOV) infection is limited. During the 2013–16 outbreak in West Africa, it was not considered epidemiologically relevant to published models or projections of intervention effects. In order to improve our understanding of the transmission dynamics of EBOV in humans, we investigated the occurrence of minimally symptomatic EBOV infection in quarantined contacts of reported Ebola virus disease cases in a recognized ‘hotspot.’

**Methodology/Principal Findings:**

We conducted a cross-sectional serosurvey in Sukudu, Kono District, Sierra Leone, from October 2015 to January 2016. A blood sample was collected from 187 study participants, 132 negative controls (individuals with a low likelihood of previous exposure to Ebola virus), and 30 positive controls (Ebola virus disease survivors). IgG responses to Ebola glycoprotein and nucleoprotein were measured using Alpha Diagnostic International ELISA kits with plasma diluted at 1:200. Optical density was read at 450 nm (subtracting OD at 630nm to normalize well background) on a ChroMate 4300 microplate reader. A cutoff of 4.7 U/mL for the anti-GP ELISA yielded 96.7% sensitivity and 97.7% specificity in distinguishing positive and negative controls. We identified 14 seropositive individuals not known to have had Ebola virus disease. Two of the 14 seropositive individuals reported only fever during quarantine while the remaining 12 denied any signs or symptoms during quarantine.

**Conclusions/Significance:**

By using ELISA to measure Zaire Ebola virus antibody concentrations, we identified a significant number of individuals with previously undetected EBOV infection in a ‘hotspot’ village in Sierra Leone, approximately one year after the village outbreak. The findings provide further evidence that Ebola, like many other viral infections, presents with a spectrum of clinical manifestations, including minimally symptomatic infection. These data also suggest that a significant portion of Ebola transmission events may have gone undetected during the outbreak. Further studies are needed to understand the potential risk of transmission and clinical sequelae in individuals with previously undetected EBOV infection.

## Author Summary

With over 28,000 reported cases, the 2013–16 West African Ebola virus disease epidemic is the largest and longest on record. This study provides further evidence that Ebola, like other viruses, causes a spectrum of clinical manifestations that may include minimally symptomatic infection. The findings also suggest that many episodes of human-to-human transmission of Ebola virus in West Africa may have gone undetected in the recent outbreak. This has implications for the definition of Ebola virus disease survivorship, delineation of transmission chains, and future vaccine studies.

## Introduction

Despite over 28,000 reported cases of Ebola virus disease (EVD) in the 2013–16 pandemic as of March 27, 2016 [1], we are only beginning to trace the complex biosocial processes that have promoted spread of the virus [2,3]. Important questions remain, including how to best use tools such as new vaccines [4] and rapid diagnostic tests [5] to contain future outbreaks, the extent to which symptomatic individuals do not present for care, how to identify and manage clinical sequelae of EVD [6], and the incidence and transmission dynamics of minimally symptomatic Ebola virus (EBOV) infection.

Evidence for minimally symptomatic EBOV infection is limited. During the 2013–16 outbreak in West Africa, it was not considered epidemiologically relevant to published models or projections of intervention effects [7–10]. Moreover, it is not known if clinical sequelae seen in survivors of EVD (e.g., uveitis) exist in individuals who had minimally symptomatic EBOV infection.

In order to improve our understanding of the transmission dynamics of EBOV in humans, we investigated the occurrence of minimally symptomatic EBOV infection in a recognized Ebola ‘hotspot’ [11], which we defined as an area with a reverse transcription polymerase chain reaction (RT-PCR)-confirmed EVD attack rate above 2% in a two-month period.

## Methods and Results

To measure the occurrence of EBOV infection in a group of people exposed to confirmed cases, we validated a commercial ELISA kit in a cohort of patients with known Ebola status and then used this tool to determine infection status in previously quarantined individuals.

### Ethics Statement

The study protocol was approved by the Sierra Leone Ethics and Scientific Review Committee and the Stanford University Institutional Review Board (Protocol ID: 33882). We held meetings with Paramount, Sectional, and Town Chiefs to discuss the proposal and supply them with written information. We then held town meetings to describe the project and answer community questions. Individuals provided written informed consent or placed a thumbprint after hearing a consent script read in the Krio or Kono languages (both ethics committees approved the use of an oral script and thumbprint for those participants who could not read or write); parents signed for children under the age of 18, and children and adolescents provided verbal assent. Subjects received 50,000 Leones (~$10 US) for their participation in the study.

### Setting

Kono is a diamond-rich district in eastern Sierra Leone which reported 301 cases of EVD (that is, RT-PCR confirmed individuals who presented for isolation or were identified by surveillance teams) between August 2014 and February 2015 (Kono District Ebola Response Centre (DERC), personal communication). Several of the authors worked closely with the Kono DERC, British military, and International Federation of the Red Cross on containment, isolation, and surveillance during the time of active transmission in the district. Parts I and II of this study were conducted from October 2015 to January 2016 by a team consisting of two physicians, a physician-anthropologist, a laboratory technician, and two community health workers.

#### I. ELISA Validation

We recruited 30 EVD survivors as positive controls by contacting the director of the Kono Survivors’ Group and requesting that she identify individuals interested in participating in a serosurvey. Using Kono DERC records, we verified that survivors had been admitted to an Ebola Treatment Unit (ETU) and had a documented RT-PCR positive result for EBOV. To identify a group of people whom we considered at very low risk of previous EBOV infection to serve as negative controls, we used Kono DERC records to locate three villages in Kono District in which no Ebola cases had been reported: In the villages of Tombodu, Wardu, and Njagbwema, we recruited 132 individuals who denied contact with Ebola cases both inside and outside of their communities.

We collected blood samples from participants in 4-mL EDTA BD Vacutainer tubes. The tubes were stored at 4C until arrival at the laboratory. Plasma was obtained by centrifugation (20 minutes, 1228g), and enzyme-linked immunosorbent assays (ELISA) were performed the day of sample collection.

We assessed anti-glycoprotein (anti-GP) and anti-nucleoprotein (anti-NP) responses to EBOV by means of commercial ELISA kits (AE 320620–1 and AE 320520–1, respectively) from Alpha Diagnostic International (ADI), San Antonio, TX, USA. These were processed according to the manufacturer’s instructions, with plasma diluted at 1:200. Optical density (OD) was read at 450 nm (subtracting OD at 630nm to normalize well background) on a ChroMate 4300 microplate reader (Awareness Technologies, Marina Del Rey, CA, USA). Values are reported in units per milliliter (U/mL) with the use of reference antibody provided by the manufacturer.

To determine overall accuracies for the two ELISAs in combination and individually, we generated receiver operating characteristic (ROC) curves and compared the areas under the curve (AUCs) for the individual and combined anti-GP and anti-NP tests [12]. The cutoff for a positive test was established by ROC analysis, defined as the point along the ROC curve that gave the highest sum of sensitivity (%) and specificity (%).

**Fig 1:**
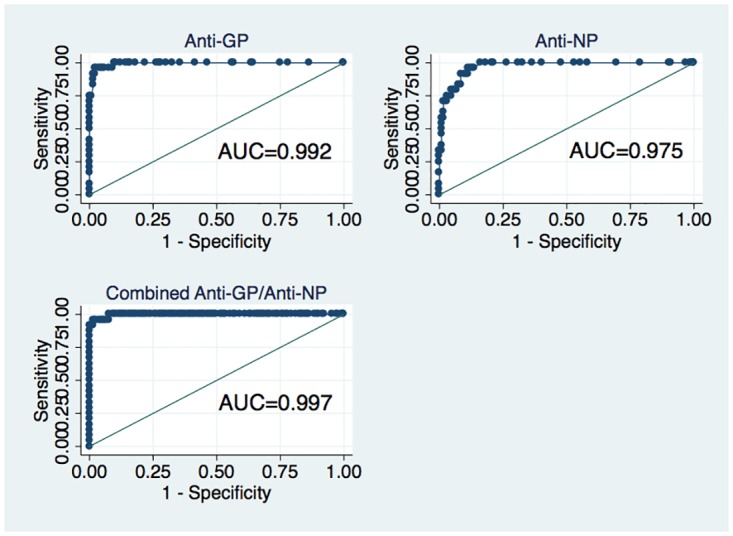
ROC curves for anti-GP, anti-NP, and a combination of the two ELISAs.

Fig 1 provides ROC curves and AUCs for the individual and combined ELISA assays. AUCs for the anti-GP, anti-NP, and combined assays were 0.992 (SE = 0.005, 95% CI = 0.983–1.000), 0.975 (SE = 0.010, 95% CI = 0.956–0.995), and 0.997 (unable to determine CIs as the combined ROC was generated through logistic regression), respectively, suggesting that these three tests were statistically equivalent. The cutoff for the anti-GP ELISA that yielded optimal sensitivity (96.7%) and specificity (97.7%) was 4.7 U/mL.

#### II. Serosurvey

**Fig 2:**
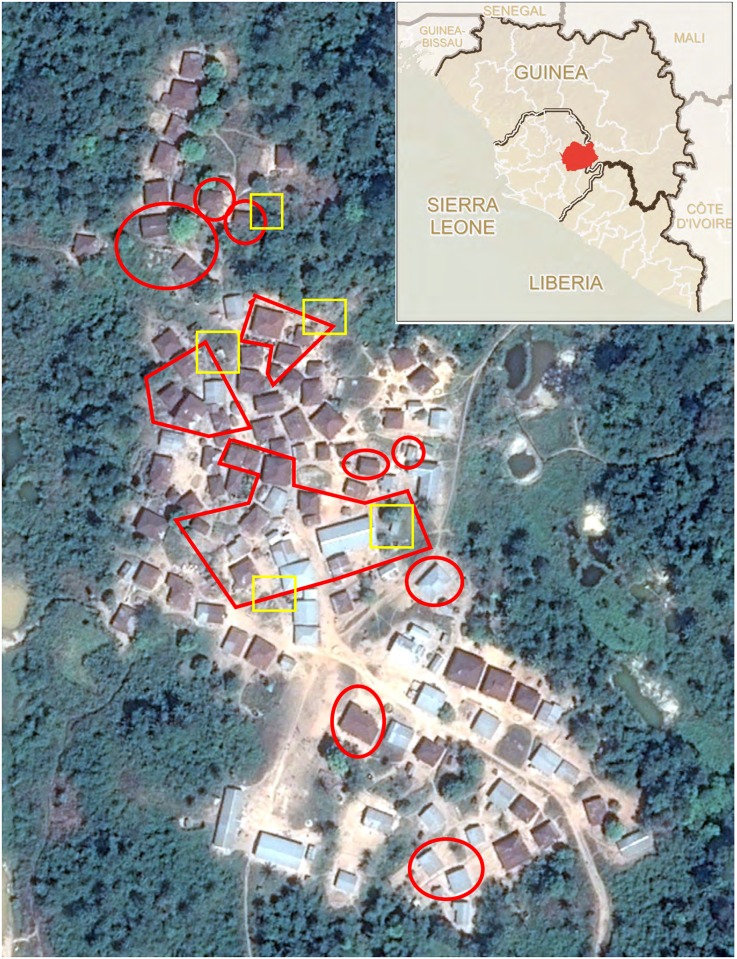
Satellite image of village. Quarantined areas are bounded in red; public latrines in yellow. Source: Google Earth Pro. (Inset: Sierra Leone and surrounds. Kono District in red. Source: Centers for Disease Control and Prevention.)

*Study population*. We conducted a serosurvey in Sukudu, a village of approximately 900 inhabitants (100 households) that was one of three major ‘hotspots’ in Kono District. We obtained a list of quarantined houses and EVD cases from the Kono DERC and verified it with the town chiefs and youth leaders. We also interviewed members of all of the affected households and discussed the identification of EVD cases with them to triangulate DERC records. We defined potential participants as having been exposed to EBOV if they stayed in a house that was quarantined based on sharing a public latrine with a confirmed case or based on a confirmed case living there. All homes were geolocated using a remotely sensed image (Fig 2).

Between December 15, 2014 and January 14, 2015, the Kono DERC reported 34 cases of EVD in Sukudu (16 confirmed cases, 12 probable cases, and 6 survivors). Probable cases were defined as any deceased suspected case having an epidemiological link with a confirmed case.

We recruited healthy children and adults age 4 and older who had been placed under quarantine during the period of active Ebola transmission in Sukudu. None of these individuals had participated in an Ebola vaccine trial. Each potential participant was screened for fever or symptoms consistent with EVD, and he/she was excluded if any were present. Medical referral to a nearby Partners In Health-supported clinic (which provides free medical care to those unable to pay) was provided.

Of 193 individuals from Sukudu who were quarantined for sharing a public latrine or living quarters with a confirmed case, only 4 declined to participate. Ten individuals were initially excluded because of fever at the time of initial contact; none of these met the case definition for EVD. On reassessment one week later, all but one had defervesced and were able to provide blood samples. Two participants had failed venipuncture attempts for blood draw. We thus sampled a total of 187 individuals for anti-GP in 24 of 25 previously quarantined houses as defined above (the members of 1 quarantined household could not be located).

*Survey*. We collected the following data from study participants: age, gender, occupation, education level, whether or not they shared a room with a confirmed case of EVD, and whether they had signs or symptoms consistent with EVD that were severe enough to prompt seeking of care during the time of their village outbreak. Specifically, these included fever or unexplained bleeding or any three of the following: headache, myalgia, rash, vomiting, diarrhea, hiccups, breathing problems, or difficulty swallowing [13]. Other household members verified signs and symptoms.

Table 1 shows that 57.8% of participants were male, 35.8% were less than 15 years of age, 48.6% worked outside the home (mostly as farmers or miners), and 79.1% had completed primary school or less. Among exposed participants who were quarantined, 50.2% shared a public latrine with a confirmed case, and 49.8% slept in the house of a confirmed case.

**Table 1:** Characteristics of research participants, Sukudu, Kono District, Sierra Leone, 2015–16.

		Total	
Characteristics		n = 187	%
**Gender**	**Male**	**108**	**57.8**
	**Female**	**79**	**42.2**
**Age**	**<15**	**67**	**35.8**
	**15–45**	**103**	**55.1**
	**>45**	**17**	**9.1**
**Occupational activity**	**Student**	**72**	**38.5**
	**Works outside the home (farming or mining)**	**91**	**48.7**
	**Housework**	**24**	**12.8**
**Highest level of school completed**	**Primary or less**	**148**	**79.1**
	**Middle school or above**	**39**	**20.9**
**Reason quarantined**	**Shared public latrine with a confirmed case**	**94**	**50.2**
	**Slept in the house of a confirmed case**	**93**	**49.8**

**Fig 3:**
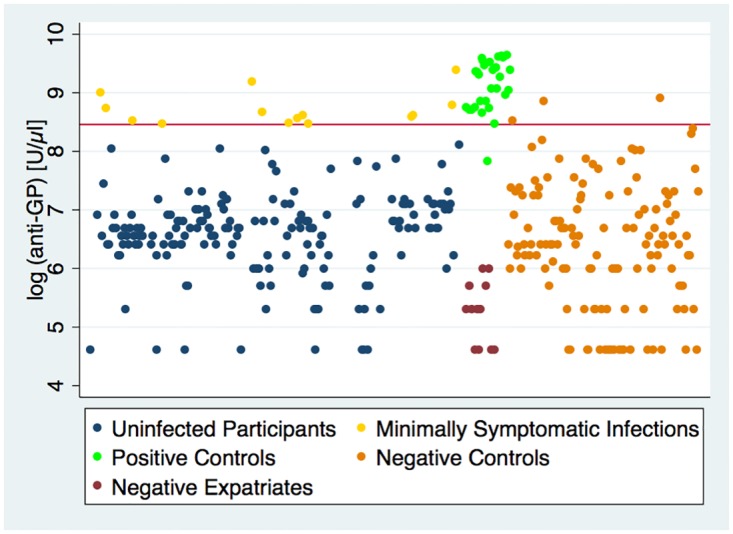
log (anti-GP) for 187 study participants, 132 negative controls, 30 positive controls, and 12 negative expatriates. The red line represents a cutoff of 4.7 U/mL [log (4700 U/μl) = 8.46], which yielded 96.7% sensitivity and 97.7% specificity in distinguishing controls.

*Laboratory analyses*. Of the 187 exposed individuals tested, none of whom were previously known to have EVD, we identified 14 (7.5%) who tested positive by anti-GP ELISA (Fig 3). Two (14.3%) of these 14 reported fever during quarantine while the remaining 12 denied any signs or symptoms during quarantine.

*Data analyses*. We considered participants to have had minimally symptomatic EBOV infection if they 1) had an anti-GP ELISA above the cut-off as determined in the validation sub-study and 2) denied symptoms consistent with EVD during the period of active transmission in the village.

In addition to the 16 confirmed EBOV infections with a fatal outcome, the 12 probable deaths from EVD, and 6 confirmed EBOV infections who survived (34 total cases), our serosurvey identified an additional 14 individuals who were anti-GP ELISA IgG-positive. Two of these individuals reported fever during the quarantine period, and 12 were classified as minimally symptomatic, contributing 25% (binomial exact 95% CI, 14% to 40%) to the total caseload. After including the 2 undocumented symptomatic cases with the 34 reported by the DERC, we calculated a 78% symptomatic case fatality ratio (binomial exact 95% CI, 61% to 90%).

## Discussion

One year after the peak of the Ebola epidemic in Sierra Leone, we identified positive IgG responses to Zaire-EBOV in 14 individuals not known to have had EBOV infection from a village classified as a hotspot for Ebola transmission. Of these, the majority reported having had no symptoms consistent with EVD during the epidemic while two reported only fever. Thus, we provide further evidence that Ebola, like many other viral infections, presents with a spectrum of clinical manifestations, including minimally symptomatic infection. In addition, our data suggest that a significant portion of Ebola transmission events may have gone undetected during the epidemic.

Although it was difficult to verify symptoms through our retrospective interviews (given the considerable denial of EVD during the outbreak due to stigma and the fear of being admitted to an ETU where those admitted were seldom discharged), our data indicate that 25% of EBOV infections may have been minimally symptomatic, which is similar to the data from empiric studies and to recent modeled estimates [14].

The phenomenon of previously undetected, minimally symptomatic EBOV infection was evident around the discovery of the virus in 1976. Using an immunofluorescence assay, the World Health Organization/International Study Team found that 19% of contacts of EVD cases—very few of whom gave any history of illness—had antibodies to the virus [15]. In 2000, Leroy and colleagues published a study (based on ELISA/Western blot) and found that of 24 asymptomatic close contacts of Gabonese patients with EVD, 11 developed both IgM and IgG responses to Ebola Zaire antigens, indicating viral infection [16]. Other investigators have found evidence of seropositive individuals in areas without large outbreaks using ELISA and postulate that there may have been active circulation of filovirus without apparent clinical manifestations [17,18]. Heffernan and colleagues also used ELISA in Gabon and found that 1% of individuals in an epidemic zone had IgG antibodies to Ebola Zaire virus, yet no history of exposure [19]. In another study in Gabon, Becquart and colleagues found a 15.3% Ebola Zaire IgG seroprevalence in 220 randomly selected villages and concluded that most of the seropositive persons identified “probably had mild or asymptomatic infection” [20]; however, they used uninfected individuals in France as negative controls. We found that unexposed expatriates (not included as negative controls) had a significantly lower mean log anti-GP (M = 5.25 U/mL, SD = 0.54, N = 12) than unexposed Sierra Leoneans (M = 6.40 U/mL, SD = 1.06, N = 132) using the two-sample t-test for unequal variances, t(20.05) = 6.40, P< = 0.001 (see Fig 3), potentially due to cross-reactivity of our assay with closely related pathogens circulating in the region.

Our study has several possible limitations. First, if asymptomatic EBOV infection is a common occurrence, it is possible that some of the “true negatives” used for the validation of our ELISA assays could have been infected with EBOV. We did not use a microneutralization assay for comparison purposes, nor did our pool of EVD survivors represent all of the possible antibody titers in the regional population. Although the serologic assay conferred sensitivity and specificity greater than 95%, the assay protocol produced antibody titers that were considered qualitative in nature. Second, we relied on study participants and their household contacts’ memories of events that had taken place up to a year prior when we classified them as symptomatic or asymptomatic (potential recall bias); however, during the outbreak, most quarantined households were monitored daily by surveillance teams that conducted symptom screens and measured temperatures, and individuals were brought to treatment facilities for EBOV testing if they screened positive. Third, our IgG assays indicate previous infection but provide no information on when that infection took place. The 3 negative controls with positive tests either represent false positives due to cross-reactive antibodies or previously infected individuals. (We did not perform IgM ELISAs, as other investigators have demonstrated that Ebola IgM titers largely diminish within 60 to 90 days of symptom onset [21,22].) Fourth, we did not ascertain whether there were non-quarantined individuals who had minimally symptomatic infection.

Our study focused on the quarantined population of one village. Extrapolation of our findings to other villages and generalizability to the epidemic should be approached with caution. Although many of the study participants were followed by surveillance teams during the time of active EBOV transmission in their village, the timing of the possible infection cannot be known from IgG data, and it is improbable that surveillance teams followed each individual throughout the duration of the outbreak in Sierra Leone. Thus, the concern that IgG positive persons indeed had but denied symptoms cannot be excluded.

Despite these limitations, our serosurvey provides a deeper perspective on EBOV transmission, and more village-level serosurveys could enhance our understanding of undetected EBOV transmission at the epidemic level. Furthermore, our findings suggest there would be value in exploring the interaction of seropositive persons and EVD cases to improve our understanding of exposure risk. As a result, we may learn more about how efforts at containment can be improved. The data also have important implications for future vaccine studies that rely on detecting antibody to EBOV. Lastly, the findings support the World Health Organization’s interim guidance on clinical care for survivors of EVD, which defines a survivor as a person:

- With a confirmed positive result on RT-PCR testing for EBOV on any body fluid who subsequently recovered;
  And/or
- Who is IgM and/or IgG positive on serological testing for EVD and has not been vaccinated against EBOV [23].

There is ongoing discussion in West Africa over the definition of survivorship, usually specified by having a positive EBOV RT-PCR result and discharge from an ETU. Plans are underway to develop national registries and provide ID cards to such survivors, so as to delineate individuals eligible for free social and medical support services. Should the notion of survivorship be extended to all those who are IgG positive, including those who had minimally symptomatic infection or who were sick but were never tested at the time of illness? How we define a community of suffering is always problematic and should be revisited given that this definition has implications for identity, stigma, and access to social and medical services.

In conclusion, by using ELISA to measure Zaire-EBOV antibody concentrations, we identified a significant number of individuals with previously undetected minimally symptomatic EBOV infection in a ‘hotspot’ village in Sierra Leone, approximately one year after the village outbreak. Further studies are needed to understand the potential risk of transmission and clinical sequelae in individuals with minimally symptomatic EBOV infection.

## Supporting Information

S1 Checklist STROBE Checklist. (DOC)
